# Soybean Root Transcriptomics: Insights into Sucrose Signaling at the Crossroads of Nutrient Deficiency and Biotic Stress Responses

**DOI:** 10.3390/plants12112117

**Published:** 2023-05-26

**Authors:** Leela Chandra Manozna Nidumolu, Kristina Mae Lorilla, Indrani Chakravarty, Claudia Uhde-Stone

**Affiliations:** Department of Biological Sciences, California State University, East Bay, Hayward, CA 94542, USA; mnidumolu@horizon.csueastbay.edu (L.C.M.N.); klorilla@gmail.com (K.M.L.); indu.chakravarty@gmail.com (I.C.)

**Keywords:** crosstalk, nutrient deficiency, sucrose signaling, transcriptome

## Abstract

Soybean (*Glycine max*) is an important agricultural crop, but nutrient deficiencies frequently limit soybean production. While research has advanced our understanding of plant responses to long-term nutrient deficiencies, less is known about the signaling pathways and immediate responses to certain nutrient deficiencies, such as P_i_ and Fe deficiencies. Recent studies have shown that sucrose acts as a long-distance signal that is sent in increased concentrations from the shoot to the root in response to various nutrient deficiencies. Here, we mimicked nutrient deficiency-induced sucrose signaling by adding sucrose directly to the roots. To unravel transcriptomic responses to sucrose acting as a signal, we performed Illumina RNA-sequencing of soybean roots treated with sucrose for 20 min and 40 min, compared to non-sucrose-treated controls. We obtained a total of 260 million paired-end reads, mapping to 61,675 soybean genes, some of which are novel (not yet annotated) transcripts. Of these, 358 genes were upregulated after 20 min, and 2416 were upregulated after 40 min of sucrose exposure. GO (gene ontology) analysis revealed a high proportion of sucrose-induced genes involved in signal transduction, particularly hormone, ROS (reactive oxygen species), and calcium signaling, in addition to regulation of transcription. In addition, GO enrichment analysis indicates that sucrose triggers crosstalk between biotic and abiotic stress responses.

## 1. Introduction

Soybean (*Glycine max*) is an important food crop in the US and worldwide, and preserving soybean crop yields is critical for the global economy and food security. Nutrient deficiencies, particularly phosphate (P_i_) and iron (Fe) deficiencies, frequently limit crop production [1,2,3]. Iron deficiency chlorosis in soybean alone results in global yield losses of millions of metric tons per year [4,5,6].

Plants need to carefully coordinate growth and development with—often scarce—nutrient availability. Glucose has been identified and thoroughly studied as an important signaling molecule of nutrient deficiency and acts by mechanisms similar to those found in yeast and mammals. Much less is known about the mechanism of sucrose signaling, which appears to be unique to plants [7]. A dual role of sucrose as both a metabolite and a signaling molecule was first suggested many decades ago [8] and has gained momentum in recent years [7,9,10,11,12].

A role for sucrose specifically in nutrient deficiency signaling has been proposed during the last two decades [13,14,15]. Split-root experiments in Arabidopsis [16] and white lupin [17,18] revealed that P_i_ deficiency responses in the root are triggered, at least in part, by low P_i_ levels of the shoot. This finding initiated a search for candidates for long-distance signals by which the shoot communicates P_i_ deficiency to the root. Independent studies identified sucrose, transported in the phloem from shoot to root, as an important long-distance signal of P_i_ deficiency [14,19]. Further studies implicated sucrose in the signaling of several other nutrient deficiencies, including iron (Fe) [15] and sulfur (S) [20]. Studies of an Arabidopsis mutant that is over-accumulating sucrose (hsp1) indicate that sucrose can act as a global regulator of plant responses to multiple nutrient deficiencies, including deficiencies of P_i_, nitrogen (N), and potassium (K) [21]. Plant responses to various nutrient deficiencies tend to overlap, a phenomenon known as crosstalk [22], which may be in part due to a shared sucrose signaling pathway.

It is pertinent to point out that the function of sucrose as an initial signal of various nutrient deficiencies is different from its re-allocation as a carbon source to the root. The latter is an acclimation to P_i_ and N deficiency that facilitates an increase in the root-to-shoot ratio under these conditions [23] and is not the focus of this study.

While it has been shown that sucrose can regulate the expression of certain genes via the activation of transcription factors such as AtMYB75 and AtWRKY20 in Arabidopsis [7,24], it is still unclear how cells receive and process the sucrose signal. Sucrose non-fermenting-1-related protein kinases (SnRKs) have been proposed to serve both as sugar transporters and sugar sensors [25], though further studies are needed to fully elucidate the potential signaling role of sugar transporters.

Significant research has been devoted to understanding plant responses to nutrient deficiencies. In recent years, transcriptomic approaches, particularly RNA-seq, have made great strides toward the identification of differential gene expression in response to various nutrient deficiencies in plants [26,27,28,29,30,31,32]. However, the underlying mechanism of sucrose signaling is not yet well understood [7,11].

In the current study, RNA-seq analysis has been applied to hydroponically grown soybeans treated with short-term (20 min, 40 min) additions of external sucrose to the roots. Our objective was to dissect the early transcriptomic responses to the signal sucrose in roots to identify potential key players in the sucrose signaling network, specifically as part of nutrient deficiency responses. A better understanding of sucrose signaling in response to various nutrient stresses could aid in developing soybeans with increased stress tolerance, productivity, and more efficient use of fertilizers.

## 2. Results

### 2.1. RNA-Seq to Assess Short-Term Responses to Sucrose Resulted in 260 Million Paired-End Reads

To mimic the increased amount of sucrose that is transported from shoot to root in response to various nutrient deficiencies, we added sucrose directly to the roots in a hydroponic system. Previous results in the legume white lupin have shown that cluster root formation, a morphological response to P_i_ and Fe deficiency, can be mimicked by external sucrose application [33]. Internal sucrose concentrations at the initiation zone of cluster root formation were measured at 3.4 mM sucrose [33]. External sucrose application increased the number of cluster roots in a concentration range of 2.5 mM to 12.5 mM sucrose; a further increase to 25 mM sucrose resulted in unusual root thickening [33]. Based on these data, we decided to add an external sucrose concentration of 10 mM, which is high enough to expect a strong response to the sucrose signal but not too high to expect non-physiological responses. After hydroponic growth for four weeks in nutrient-sufficient conditions, roots were subjected to external sucrose for 0 min (control), 20 min, and 40 min in three biological replications each, for a total of nine samples.

The nine pooled, bar-coded cDNA libraries were sequenced using the Illumina NovaSeq6000 150PE Flow Cell SP platform, resulting in an initial 485.64 million total reads of 150 bp each. FastQC quality checks revealed overall very good sequence quality, with Phred quality scores above 30 even before trimming, except for the first two and last three nucleotides. A quality check after trimming showed Phred quality scores above 30 for all positions, complete adaptor removal, and a total number of paired sequences of 260 million (Table 1).

We used HiSat2 [34] and StringTie [35] to map our paired sequence reads to the *G. max* reference genome [36], which has been reannotated in 2021 (RefSeq assembly accession: GCF_000004515.6_Glycine_max_v4.0; annotation date 3 October 2021). Our mapping resulted in a total of 62,048 mapped genes, which is more than the currently annotated 59,046 genes in GCF_000004515.6_Glycine_max_v4.0, according to the corresponding gene and feature statistics for this annotation (release 104, https://www.ncbi.nlm.nih.gov/genome/annotation_euk/Glycine_max/104/ (accessed on 14 October 2022)).

Some of these novel genes were found at a very low level, raising the question if they are real. When setting a threshold of at least 3 total reads, we found 61,675 genes, which still includes some novel genes. Applying the same threshold of at least 3 total reads, StringTie identified 99,775 different transcripts, indicating alternative splicing.

### 2.2. Short-Term Sucrose Exposure Changes the Expression of Hundreds of Genes

**Figure 1:**
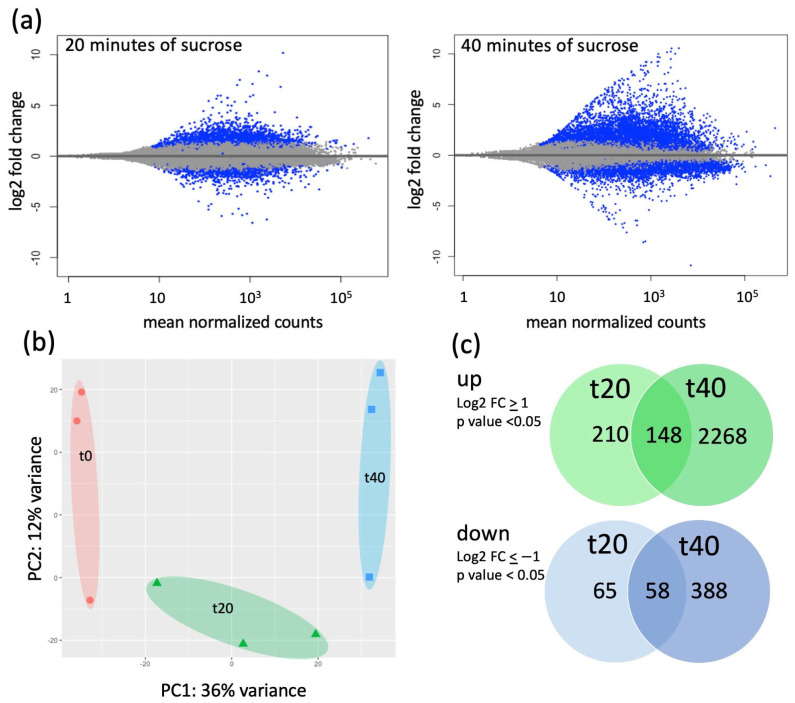
(**a**) MA (mean average) plot of log2 FC against normalized sequence counts at 20 min (t20) and 40 min (t40) of sucrose treatment, each compared to t0 (no-sucrose control). To reduce background noise, shrinkage of the effect size was applied before visualization. Values of padj (adjusted *p*-value) ≤0.01 in the DESeq2 gene expression analysis are shown in blue. (**b**) PCA plot of the three biological replications representing differences between both time points (20 min, 40 min) and control (t0). (**c**) Venn diagram: significantly up-regulated DEGs in t20 and t40 with a *p*-value < 0.05 and a log2 FC ≥ 1 are represented on top (green), and significantly down-regulated genes in t20 and t40 with a *p*-value < 0.05 and a log2 FC ≤ 1 are represented on the bottom (blue).

MA (mean average) plots (Figure 1a) were used to visualize log2 FC (fold change) against normalized sequence counts at 20 min and 40 min of sucrose exposure, each compared to 0 min (control). The plot reveals some changes in expression (blue dots) at 20 min and more drastic changes after 40 min of sucrose exposure. A PCA (principal component analysis) plot (Figure 1b) displays clear differences between the three time points used in this study (t0, t20, and t40), though it also reveals some variability between the biological replicates.

We extracted normalized expression data (FPKM, fragment per kilobase, and million) from our HiSat2 data using Ballgown [35,37]. A Venn diagram (Figure 1c) represents the total number of up- and down-regulated genes in response to 20 min and 40 min of sucrose treatment. A total of 148 upregulated and 58 downregulated genes are shared between 20 min and 40 min of sucrose responses.

### 2.3. Genes Involved in Auxin-Mediated Responses and Nutrient Assimilation Are among the Most Highly Upregulated Genes in Response to Sucrose

**Figure 2:**
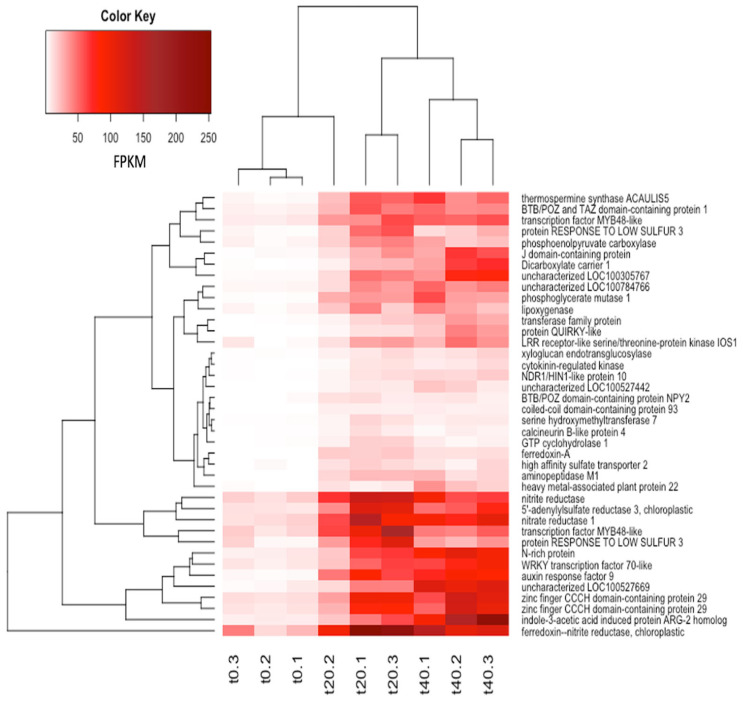
Heatmap of the top 40 up-regulated genes in both t20 and t40 (sorted for t20) across biological replications. Shown are the relative numbers of normalized reads in FPKM for the three biological replications: t0 (control), t20, and t40.

A heatmap (Figure 2) gives an overview of normalized expression (in FPKM) for the top 40 up-regulated genes after 20 and 40 min of sucrose treatment, revealing low expression levels without sucrose and increased expression at both 20 and 40 min of sucrose.

Table 2 shows the 10 most highly upregulated genes in response to 20 min of sucrose. The *description* column indicates the involvement of upregulated genes in various functions, including auxin signaling, nitrogen and sulfur assimilation, and transcriptional regulation. A heatmap of the 20 most downregulated genes in response to sucrose is shown in Figure S1.

### 2.4. Gene Ontology Analysis Reveals High Proportions of Transcription Factors and Internal Signaling in Response to Sucrose

**Figure 3:**
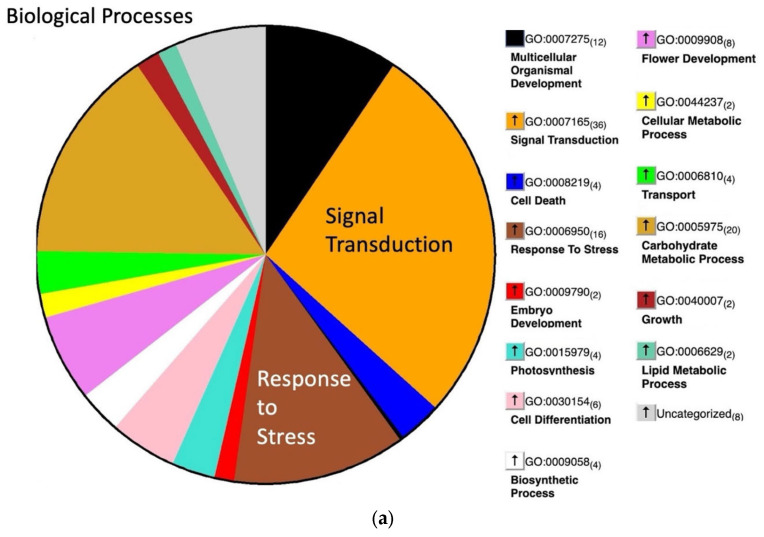

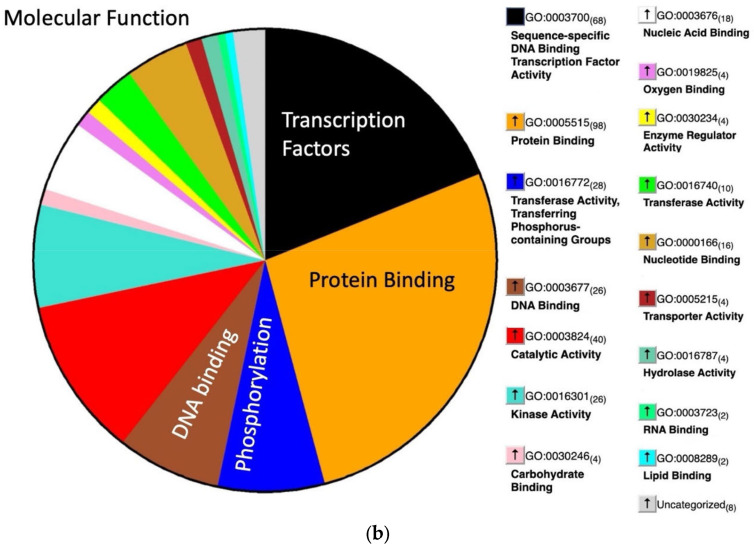
GO analysis of biological processes (**a**) and molecular functions (**b**) among highly upregulated genes (Log2FC > 3, *p*-value < 0.05) at t40.

An analysis of GO (gene ontology) terms indicates a high proportion of sucrose-induced genes belong to the categories of *signal transduction* and *stress response* (Figure 3a). The largest two groups among *molecular functions* are *protein binding* and *transcription factors* (Figure 3b). Because our GO analysis of *molecular functions* revealed a high proportion of transcription factors (TFs), we took a closer look at this group of genes. Our RNA-seq analysis revealed a total of 143 transcription factors (TFs) that were differentially expressed (log2FC > 1.5, *p*-value < 0.5 or <−1.5, *p*-value < 0.5) in response to sucrose; the majority of these (128) were upregulated, while 15 were downregulated.

At 20 min of sucrose treatment, only nine TFs were significantly upregulated, and only two of these (WRKY 70-like and UNE12) were also upregulated after 40 min of sucrose. In contrast, a total of 121 TFs were upregulated at 40 min of sucrose treatment. In terms of frequency, this means that at t20, 2.5% of upregulated genes were transcription factors, while at t40, this number increased to 5%. Table 3 shows the ten most highly upregulated transcription factors in response to 40 min of sucrose. While only t40 shows significant *p*-values, the t20 fold changes indicate that the trend of upregulation of these transcription factors starts earlier. The most frequent families among upregulated TFs are WRKY (33 TFs), ethylene response factors (ERFs, 24 TFs), MYB (22 TFs), and basic helix-loop-helix (bLHL, 14 TFs).

### 2.5. GO Enrichment Indicates Involvement of ROS and Ca^2+^ Signaling in Responses to Sucrose

Enrichment analysis of GO terms displayed no significantly enriched categories at t20 but highly enriched categories at t40. Table 4 shows the fifteen most highly enriched categories at 40 min of sucrose exposure. The three most highly enriched categories.

The response to chitin, respiratory burst, and hypersensitive response all indicate a strong biotic stress response induced by 40 min of sucrose. Interestingly, ROS production (as in respiratory burst) can also be involved in abiotic stress responses [47].

To further analyze the possible involvement of ROS signaling in response to sucrose, we took a closer look at class III peroxidases (PERs), which can be involved in signaling pathways in response to both biotic and abiotic stresses [48]. We found no PERs significantly induced at 20 min of sucrose treatment. However, at 40 min of sucrose, 18 PERs were significantly upregulated; the ten most highly upregulated peroxidase genes are shown in Table S1. ROS signaling, in turn, can involve calcium-binding proteins and kinases. GO terms related to calcium signaling, such as Ca^2+^ binding, were enriched among genes upregulated after 40 min of sucrose treatment (corrected *p*-value 0.0001). We identified 45 genes putatively involved in Ca^2+^ signaling that were upregulated at t40, six of which were already upregulated at t20; the ten most highly upregulated genes in this category are shown in Table S2.

## 3. Discussion

### 3.1. RNA-Seq to Unravel the Regulatory Network in Response to Sucrose

Sucrose can act both as a signal and as a carbon source. Sucrose, transported via phloem from the shoot to the roots, has been identified as a long-distance signal for various nutrient deficiencies, including P_i_ [13,14], Fe [15], sulfur [20], and nitrogen [21]. It is worth pointing out that sucrose signaling is different from sucrose being reallocated as a carbon source, which is found only under certain nutrient deficiencies (P_i_ and N) [23] and is not addressed in our study.

RNA-seq proved useful as a high-throughput method to look at the global effect of sucrose on gene expression in order to unravel the regulatory network that is activated by the increase of sucrose in roots. Our results reveal a complex regulatory network, including a high proportion of transcription factors and members of signal transduction pathways, which we will discuss below in more detail.

As we are particularly interested in key regulators, we analyzed our data for differentially expressed transcription factors. Of the transcription factors upregulated in this study, WRKY, ERFs (ethylene response factor), and MYB were the three largest groups. Members of these families of transcription factors are known to play critical roles in regulating plant growth, development, and stress responses. Several studies have shown that members of these families of transcription factors are involved in mediating sucrose signaling in plants. AtWRKY20 and AtMYB75 have been identified as upstream regulators controlling sucrose-responsive genes (reviewed by [7]). Our study identified several transcription factors induced after 20 min and more than hundred transcription factors induced after 40 min of sucrose, providing insights into the regulatory networks that govern plant responses to sucrose signaling. We found that the most frequent families among upregulated TFs are WRKY, ethylene response factors, MYB14, MYB30, WRKY41, and WRKY33, and basic helix-loop-helix, with MYB and WRKY representing the most highly upregulated transcription factors.

### 3.2. ROS and Ca^2+^ Signaling Act Downstream of Sucrose

GO (gene ontology) analysis revealed that genes involved in the production of reactive oxygen species and intracellular signaling were highly enriched categories. Several class III peroxidases, calcium-dependent protein kinases (CDPKs), and calcium-binding proteins were upregulated after 20 min, and many more after 40 min of sucrose treatment. ROS signaling is a known response to biotic and abiotic stresses, such as salinity, heavy metals, and nutrient deficiency [47,49]. ROS can be generated by various enzymatic pathways, including NADPH oxidases and class III peroxidases, which can act as ROS producers or consumers [50]. ROS functions in concert with other important second messengers, specifically Ca^2+^, which in turn is part of Ca^2+^ signaling via activation of calcium-dependent protein kinases (CDPKs) and calcium-binding proteins, such as calmodulins [51,52]. The fact that some class III peroxidases and proteins involved in Ca^2+^ signaling were upregulated after 20 min and many more after 40 min of sucrose treatment suggests that both ROS (reactive oxygen species) and Ca^2+^ signaling pathways are triggered by sucrose and thus act downstream of sucrose signaling.

### 3.3. Sucrose Activates Plant Hormone Signaling

The two most highly upregulated genes at 20 min of sucrose exposure were auxin response factor 9 (ARF9, an auxin-induced transcriptional regulator) and aminopeptidase M1, which has been shown to be involved in polar auxin transport [39]. Auxin is known as an important mediator acting downstream of sucrose in response to multiple nutrient deficiencies. For example, increased sucrose accumulation has been shown to regulate Fe-deficiency responses in Arabidopsis by promoting auxin signaling [15].

Salicylic acid and jasmonic acid signaling were among the significantly enriched categories at 40 min of sucrose. Both hormones are known to play important roles in ROS signaling, as they are involved in the regulation of ROS production and scavenging in response to biotic and abiotic stresses [53]. These data indicate that plant hormone signaling, particularly auxin, jasmonic acid, and salicylic acid, is involved in the immediate plant responses to sucrose.

### 3.4. Sucrose May Mediate Crosstalk between Biotic and Abiotic Stresses

Crosstalk is the somewhat surprising phenomenon of plants not only responding to a specific stress but also to other stresses they do not experience at this point [54,55,56]. Because sucrose is a signal of various abiotic stresses, including multiple nutrient deficiencies [21], we speculate that sucrose may be responsible for the reported cross-talk between plant responses to various nutrient deficiencies. Supporting this hypothesis, we found several genes involved in nitrogen and sulfur assimilation among our top upregulated genes in response to 20 min of sucrose. In addition, our findings reveal that sucrose may also mediate crosstalk between biotic and abiotic stresses, as genes involved in both stress types were enriched among sucrose-responsive genes.

Taken together, our results provide insights into transcriptional regulators, ROS- and Ca^2+^-dependent signaling, as well as hormonal responses, that are all activated by sucrose. In the future, it will be interesting to look at even earlier time points to find the earliest key regulators responding to sucrose signaling. In addition, quantitative phosphoproteomics could be used to identify members of the signal transduction pathways activated in response to sucrose, as some of these are differentially phosphorylated rather than differentially expressed.

## 4. Materials and Methods

### 4.1. Seed Germination, Treatments, and Harvest

*Glycine max* cv. Williams 82 seeds (friendly gift from John Harada and Julie Marie Pelletier, UC Davis) were sterilized by shaking for 4 min in 10% bleach, followed by 6 rinses with autoclaved water. Agar plates were prepared with 0.6% agarose in petri dishes, and seeds were placed on the agar plates, covered with aluminum foil, and incubated at 27 °C. After about 4 days, once the radicle reached 3 to 5 cm in length, germinated seeds were transferred to containers containing about 850 mL of half-strength Hoagland nutrient solution [57] and were grown for 4 weeks; the Hoagland solution was changed every 4 days. The growth chamber conditions were maintained at ~27 °C with a light cycle of 16 h [58]. For sucrose treatment, 8.5 mL of 1 M sucrose (prepared in a half-strength Hoagland solution) was added directly to the hydroponic solution for a final concentration of 10 mM sucrose.

Harvesting occurred at 0 min (control) and after 20 min and 40 min of sucrose addition. To enable statistical data analysis, all-time points were performed in 3 biological replications (3 plants). Per plant, about 100 mg of root tip sections (~2 cm) were harvested in liquid nitrogen and immediately stored at −80 °C.

### 4.2. RNA Isolation and Quality Check

RNA from frozen root samples was isolated using the RNeasy Plant Mini kit (Qiagen), following the protocol for “Purification of Total RNA from Plant Cells and Tissues, and Filamentous Fungi”. The Qubit 4 Fluorometer (Thermofisher Scientific, Waltham, MA, USA) was used in conjunction with RNA-High Sensitivity and RNA IQ assays to assess both the quantity and quality of each sample. To assess quality, the Qubit RNA IQ assay represents the ratio of large and/or structured RNA to small RNA in the sample. A low score indicates that the sample consists mainly of small RNA, while a high score indicates that the sample consists mainly of large and/or structured RNA. An RNA IQ score of 8 or higher represents good quality RNA; we only used samples with an RNA IQ score of 8 or greater for RNA sequencing.

### 4.3. cDNA Library Preparation and RNA-Sequencing

Using the Stranded mRNA Kit (Illumina, Foster City, CA, USA), we converted the extracted RNA to cDNA, following the manufacturer’s instructions. Unique dual barcoding for each cDNA library was performed using the IDT for Illumina RNA UD Indexes Set A (Illumina).

Quantity and quality of cDNA libraries were assessed via TapeStation (Agilent, Santa Clara, CA, USA), and remaining adaptors were cleaned up where necessary using AMPure XP magnetic beads (New England Biolabs, Ipswich, MA, USA at a ratio of 1 volume of DNA to 0.8 volumes of beads.

Exact quantification, pooling, and sequencing of the nine barcoded cDNA libraries were performed by QB3 (the Institute for Quantitative Biosciences at UC Berkeley, CA, USA). Sequencing of the pooled cDNA libraries was performed on one flowcell lane on the Illumina Nova-Seq6000 150PE (paired-end) next-generation sequencing platform.

### 4.4. RNA-Seq Data Analysis

Illumina Conversion Software bcl2fastq2 (v2.20) was used to demultiplex the obtained sequencing data and to convert base call files into FASTQ files.

We transferred demultiplexed FastQ files containing 485.64 million reads from the QB3 server to our storage allocation at the EXPANSE supercomputer housed at SDSC (San Diego Supercomputer Center, CA USA). We checked sequence quality using FastQC (https://qubeshub.org/resources/fastqc, accessed on 1 August 2022). Next, we removed adaptors and any low-quality sequences with TRIMMOMATIC Version 0.32 [59]. The quality of the sequences was again checked by FastQC to ensure TRIMMOMATIC properly removed all adaptors and low-quality regions. Then all paired RNA-seq reads were mapped with HiSat2 [34] to the *G. max* reference genome [36], which has been reannotated in 2021 (RefSeq assembly accession: GCF_000004515.6_Glycine_max_v4.0, annotation date 3 October 2021). After initial mapping, we used StringTie [35] for transcript assembly.

### 4.5. Differential Expression Analysis

We used the prepDE.py3 script (http://ccb.jhu.edu/software/stringtie/dl/prepDE.py3, accessed on 20 October 2022) to extract two csv (comma separated value) files with transcript and gene count information. These files were explored further in RStudio. The normalized expression data FPKM (fragment per kilobase and million) was extracted using the R package Ballgown, which allows spliced transcriptome assembly for differential expression analysis [37]. To include DEGs (differentially expressed genes) with expression below detection under some conditions (circumventing the problem of not being able to divide by 0), a small FPKM value of 1 was added to all FPKM data points.

To identify DEGs, we set a threshold of log2FC (fold change) ≥ 1.5 or ≤1.5 and a *p*-value < 0.05. The corresponding LOC gene IDs were mapped to GLYMA IDs using the rentrez package (Entrez in R, v. 1.2.3; http://cran.nexr.com/web/packages/rentrez/ accessed on 2 November 2022), which is an R-based package provided by NCBI. The terms were mapped by querying the LOC IDs in the NCBI gene database, then parsing the GLYMA IDs from the locus tag of the gene from its summary.

DESeq2 was used to generate MA (mean average) and PCA (principal component analysis) plots [60]. Heatmaps of FPKM data were generated by gplots (https://cran.r-project.org/web/packages/gplots/index.html, accessed on 15 November 2022). Enrichment analysis of GO terms was performed on DEGs using the GO term enrichment tool (https://www.soybase.org/tools.php, accessed on 2 March 2023) on SoyBase [61,62].

## 5. Conclusions

In conclusion, our RNA-seq analysis revealed that short-term exposure of roots to sucrose activates multiple signaling pathways, including ROS, Ca^2+^, and hormone signaling. As sucrose induces responses to both biotic and abiotic stresses, we further conclude that sucrose acts as a mediator of crosstalk between biotic and abiotic stresses. Understanding the mechanisms of sucrose signaling in plants is important for developing strategies to improve plant productivity under biotic and abiotic stresses.

## Figures and Tables

**Table 1:** Number of paired sequences for each of the nine cDNA libraries.

cDNA Library, Biological Replicate (Rep)	Number of Paired Sequences (in Millions)	Overall Mapping Alignment Rate
Control (t0), rep 1	24.9	94.96%
Control (t0), rep 2	26.5	89.61%
Control (t0), rep 3	40.6	93.34%
20 min sucrose (t20), rep 1	24.2	92.29%
20 min sucrose (t20), rep 2	47.3	91.00%
20 min sucrose (t20), rep 3	33.1	93.34%
40 min sucrose (t40), rep 1	19.5	87.91%
40 min sucrose (t40), rep 2	26.7	81.77%
40 min sucrose (t40), rep 3	16.9	80.44%

**Table 2:** Ten most highly upregulated genes after 20 min (t20) and 40 min (t40) of sucrose, sorted for t20.

Annotation/ *G. max* ID (if Available)	Description	t20		t40	
		Log2FC	*p*-Value	Log2FC	*p*-Value
**auxin response factor 9** (**ARF9**)/transcriptional regulator	potential mediators of auxin signaling in response to biotic and abiotic stress [38]	4.3	0.007	4.7	6.92 × 10^−5^
**aminopeptidase M1**/ GLYMA.04G053300	metallopeptidase, involved in polar auxin transport [39] and root hair development [40]	4.2	0.003	4.0	0.015
**ferredoxin-A**/ GLYMA.05G168400	in roots, part of nitrogen assimilation via ferredoxin-nitrite reductase (NiR) [41]	4.2	0.00002	3.5	6.76 × 10^−6^
**phosphoglycerate mutase 1**	enzyme of glycolysis, induced by sucrose and auxin [42]	4	0.00016	4.4	0.014
**RESPONSE TO LOW SULFUR 3**/GLYMA.04G225500	function still unknown; possible transcriptional regulator involved in plant responses to environmental challenges [20]	3.7	0.041	2.6	0.023
LOC100305767/ GLYMA.08G158100	uncharacterized	3.6	0.043	4.6	0.014
**thermospermine synthase** ACAULIS5/ GLYMA.14G099200	involved in the synthesis of thermospermine, which may act as a plant growth regulator [43]; some thermospermine synthases are regulated by plant stress hormones [44]	3.6	0.020	3.9	0.005
**Dicarboxylate carrier 1**, transporter of organic acids	may shuttle malate between the cytosol and mitochondria; induced in Fe-deficient roots [45]	3.5	0.049	5.1	0.012
**J domain**-containing protein/ GLYMA.08G074200	co-chaperones of Hsp70s (heat-shock proteins), likely involved in growth, development, and stress response [46]	3.2	0.045	4.3	0.014
**RESPONSE TO LOW SULFUR 3**/ GLYMA.06G139300	function still unknown; possible transcriptional regulator involved in plant responses to environmental challenges [20]	3.2	0.049	2.0	0.013

**Table 3:** Ten most highly upregulated transcription factors (log2FC > 1.5, *p*-value < 0.5, indicated in bold) at t40. Data for t20 are also shown; while not statistically significant, some trends of upregulation can already be observed at 20 min of sucrose exposure.

Annotation	*G. max* ID (if Available)	t20		t40	
		log2FC	*p*-Value	log2FC	*p*-Value
MYB14	GLYMA.06G300200	3.8	0.099	**6.3**	**0.018**
WRKY41	GLYMA.05G215900	4.6	0.054	**5.7**	**0.007**
MYB30	GLYMA.06G300100	2.4	0.112	**5.6**	**0.034**
WRKY33	GLYMA.03G042700	3.2	0.076	**5.1**	**0.003**
MYB30	GLYMA.12G104800	2.4	0.114	**4.6**	**0.015**
WRKY SUSIBA2		2.4	0.103	**4.5**	**0.002**
ERF 13	GLYMA.03G111700	1.2	0.116	**4.1**	**0.045**
MYB13	GLYMA.12G104600	1.3	0.118	**4.1**	**0.045**
ERF098-like	GLYMA.10G036600	1.3	0.179	**3.9**	**0.00003**
WRKY40	GLYMA.07G023300	2.1	0.073	**3.7**	**0.017**

**Table 4:** Top fifteen enriched GO categories among genes upregulated at 40 min of sucrose treatment.

GO Term	Expressed GO	Expected Expression	Genome GO Count	Corrected *p*-Value *
response to chitin	183	51	1135	4 × 10^−50^
respiratory burst involved in defense response	91	19	420	1.8 × 10^−34^
regulation of plant-type hypersensitive response	122	46	1019	7 × 10^−21^
protein targeting the membrane	122	46	1020	7.6 × 10^−21^
salicylic acid-mediated signaling pathway	76	21	458	7.9 × 10^−21^
negative regulation of programmed cell death	82	24	525	8.4 × 10^−21^
intracellular signal transduction	76	22	479	1.3 × 10^−19^
response to wounding	119	46	1031	5.1 × 10^−19^
negative regulation of the defense response	97	34	766	5 × 10^−18^
MAPK cascade	78	26	575	5.6 × 10^−16^
ethylene biosynthetic process	50	12	270	2.7 × 10^−15^
jasmonic acid-mediated signaling pathway	94	36	800	3 × 10^−15^
defense response to fungus	104	42	941	4.7 × 10^−15^
systemic acquired resistance	85	31	688	5.5 × 10^−15^
salicylic acid biosynthetic process	79	29	653	2.56 × 10^−13^

* Bonferroni correction (Fisher test *p*-value multiplied by number of GO terms in gene list of interest).

## Data Availability

The raw data and processed FPKM data for all samples of this RNA-seq experiment are openly accessible through the NCBI GEO Series accession number GSE228888. (https://www.ncbi.nlm.nih.gov/geo/query/acc.cgi?acc=GSE228888, submitted 3 April 2023).

## Supplementary Materials

The following supporting information can be downloaded at: https://www.mdpi.com/article/10.3390/plants12112117/s1, Figure S1: Heatmap of the 40 most down-regulated genes after 20 min and 40 min of sucrose treatment (sorted for t20) across biological replications, Table S1: Ten most highly upregulated class III peroxidases after 40 min of sucrose treatment, Table S2: Ten most highly upregulated genes involved in calcium signaling after 40 min of sucrose treatment.
