# Supplementary material for: Soybean Root Transcriptomics: Insights into Sucrose Signaling at the Crossroads of Nutrient Deficiency and Biotic Stress Responses
Source: Plants (Basel). 2023 May 26;12(11):2117. doi: 10.3390/plants12112117 (PMC10255639; doi:10.3390/plants12112117)
Supplement: Supplementary file 1 [file plants-12-02117-s001.zip › Soybean_RNA-seq_Supplementary_Materials.pdf]

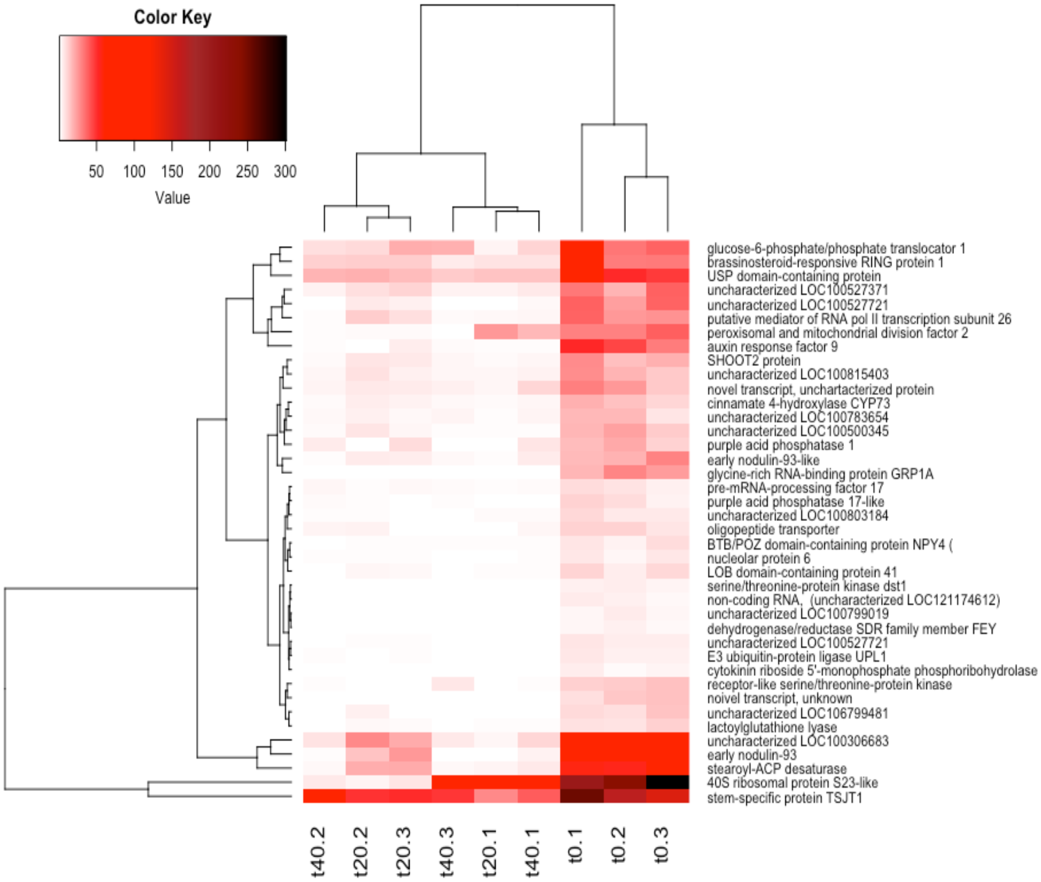

Figure S1. Heatmap of the 40 most down-regulated genes after 20 min and 40 min of sucrose treatment (sorted for t20) across biological replications. Shown are the relative numbers of normalized reads in FPKM for the three biological replications each of t0(control), t20 and t40.

Table S1. Ten most highly upregulated class III peroxidases (log2FC >1.5, pvalue <0.5, indicated in bold) after 40 min of sucrose treatment. Data of t20 are also shown, though not significant.

**Annotation**	***G. max* ID** *(if available)*	**t20**		**t40**	
		**log2FC**	**pvalue**	**log2FC**	**pvalue**
peroxidase 5	GLYMA.13G106400	3.6	0.273	**3.6**	**0.013**
cationic peroxidase 1	GLYMA.02G233900	2.3	0.108	**3.5**	**0.035**
peroxidase 5	GLYMA.20G001400	1.9	0.251	**3.3**	**0.006**
peroxidase 1	GLYMA.20G241800	3.1	0.245	**3.3**	**0.012**
cationic peroxidase 2	GLYMA.09G284700	1.9	0.249	**2.8**	**0.047**
peroxidase 5	GLYMA.09G277800	-0.2	0.680	**2.7**	**0.019**
peroxidase 4-like	GLYMA.08G179700	1.3	0.022	**2.7**	**0.005**
peroxidase 52-like	GLYMA.10G191700	0.2	0.411	**2.3**	**0.022**
peroxidase 5	GLYMA.14G201700	1.5	0.121	**2.2**	**0.026**
peroxidase 5	GLYMA.06G145300	1.4	0.232	**1.9**	**0.005**

Table S2. Ten most highly upregulated genes involved in calcium signaling (log2FC >1.5, pvalue <0.5, indicated in bold) after 40 min of sucrose treatment. Data of t20 are also shown, and where statistically significant are shown in bold.

**Annotation**	***G. max* ID *(if available)***	**t20**		**t40**	
		**log2FC**	**pvalue**	**log2FC**	**pvalue**
**calcium-binding protein CML19**	**GLYMA.17G128900**	**2.5**	**0.07**	**4.2**	**0.019**
**calcium-binding protein**	**GLYMA.04G136200**	**2.5**	**0.05**	**4.1**	**0.023**
**calcium-binding protein CAST**	**GLYMA.09G270900**	**1.8**	**0.09**	**4.0**	**0.003**
calcium-transporting ATPase 2, plasma membrane-type	GLYMA.11G048300	1.3	0.17	**3.8**	**0.009**
calmodulin-like protein	GLYMA.10G002200	2.0	0.122	**3.8**	**0.0086**
**calcium-binding protein PBP1-like**	**GLYMA.13G283600**	**3.7**	**0.08**	**3.6**	**0.008**
**calcium-binding protein CML46**	**GLYMA.18G260700**	**1.7**	**0.02**	**3.4**	**0.023**
calmodulin calcium-dependent NAD kinase	GLYMA.10G185200	1.1	0.14	**3.4**	**0.003**
calcium-dependent protein kinase 28	GLYMA.01G166100	1.4	0.09	**3.0**	**0.00004**
**calmodulin-binding receptor-like cytoplasmic kinase 2**	**GLYMA.11G178300**	**2.1**	**0.041**	**3.0**	**0.0009**
